# New Appliances and Things Medical

**Published:** 1895-05-04

**Authors:** 


### NEW APPLIANCES AND THINGS MEDICAL.

[We shall be glad to receive, at our Office, 428, Strand, London, W.C., from the manufacturers, specimens of all new preparations and appliances which may be brought out from time to time.]

### HOME-MADE BEEF TEA, ESSENCE OF BEEF, CONCENTRATED BEEF TEA.

(Frederick Mason, York Works, Acre Lane, Brixton, S.W.)

These preparations have for many years successfully stood the test of hospital and family use. The "Home-Made Beef Tea" is of double the ordinary strength, and may be recommended as most palatable, in which respect it should be especially welcome to the invalid in contrast to the greasy and frequently badly-prepared beef tea procured from the kitchens of many households. The "Essence of Beef" and "Concentrated Beef Tea" are so prepared as to contain only the most stimulating and exhilarating properties of the meat, and may be taken with safety in cases of difficult digestion. Economy in price and the fact that, being hermetically closed in air-tight tins, they will keep in any climate should constitute a further recommendation to these wholesome, palatable, and stimulating preparations.

### CALVERT'S "DOMESTIC" CARBOLIC VAPORISER.

This is a tin soup-plate shaped holder standing on a tin-plate receptacle, which also supports a "fairy light" night-light. A sufficiency of No. 4 carbolic acid or 50 per cent. carbolic powder is placed in the hollow plate, and the night-light started. A slow evolution of carbolic fumes ensues, and the whole arrangement lends itself to the very efficient vaporising of the acid. It will be found very convenient and useful in all cases where carbolic acid is required to be free in the atmosphere, as in sick rooms where infectious disease is being treated, or where insect pests are to be got rid of.

